# Supplementary material for: Patients with anxiety disorders rely on bilateral dlPFC activation during verbal working memory
Source: Soc Cogn Affect Neurosci. 2020 Nov 4;15(12):1288–98. doi: 10.1093/scan/nsaa146 (PMC7759210; doi:10.1093/scan/nsaa146)
Supplement: nsaa146_Supp [file nsaa146_supp.zip › scan-20-198-File006.docx]

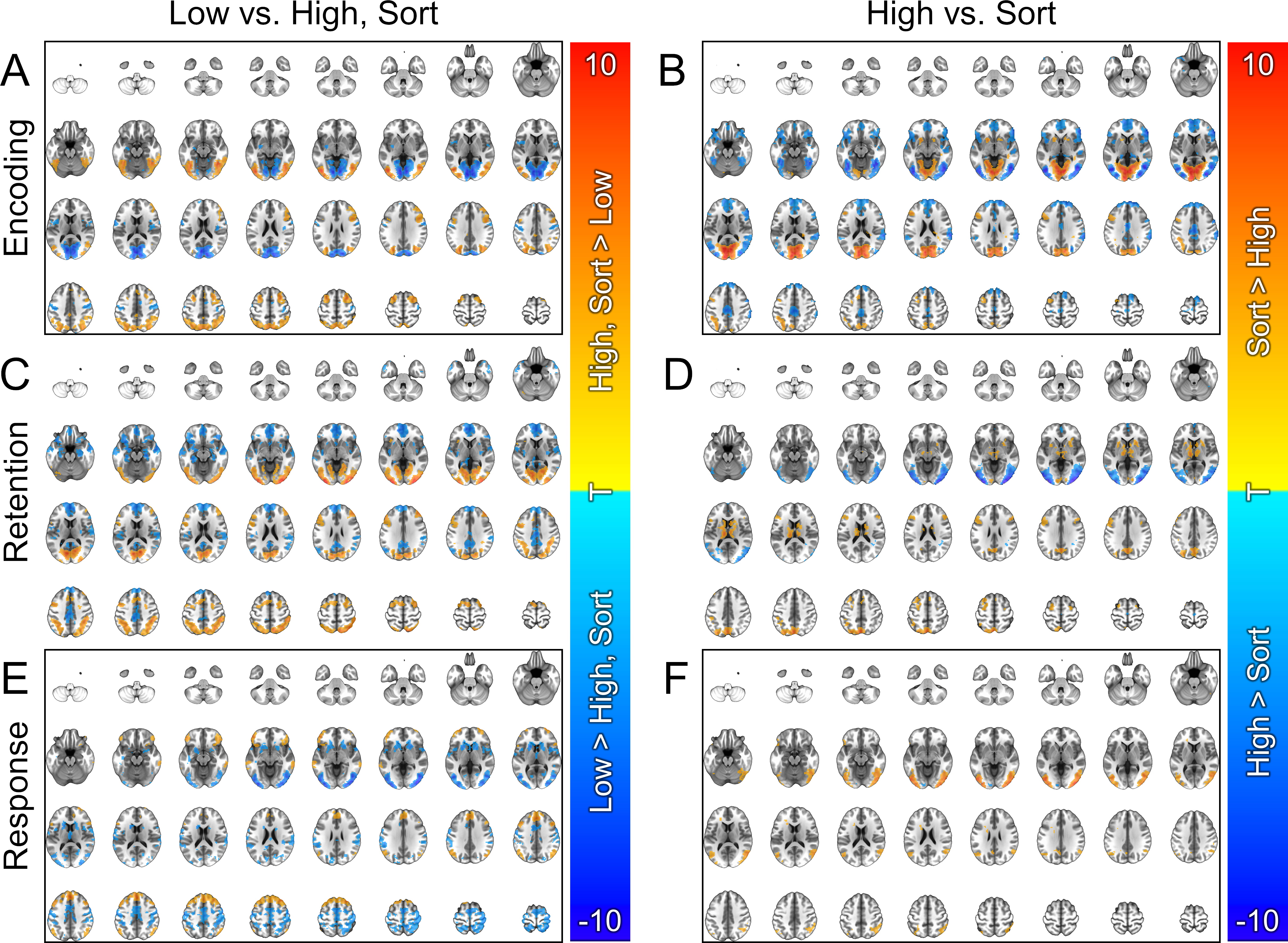


**Supplemental Figure 1: Whole brain fMRI results.** Voxelwise results for the encoding (**A, B**) retention (**C, D**), and response (**E, F**) intervals for the low vs. high, sort (**A, C, E**) and high vs. sort comparisons (**B, D, F**). Colors represent voxelwise t-test results from the planned comparisons.
