# Supplementary material for: Patients with anxiety disorders rely on bilateral dlPFC activation during verbal working memory
Source: Soc Cogn Affect Neurosci. 2020 Nov 4;15(12):1288–98. doi: 10.1093/scan/nsaa146 (PMC7759210; doi:10.1093/scan/nsaa146)
Supplement: nsaa146_Supp [file nsaa146_supp.zip › scan-20-198-File001.docx]

**Title**: Anxiety patients rely on bilateral dlPFC activation during verbal working memory.

**Running title:** Bilateral dlPFC activity in anxiety patients.

**Authors:** Nicholas L. Balderston^1,2^, Elizabeth Flook^1^, Abigail Hsiung^1^, Jeffrey Liu^1^, Amanda Thongarong^1^, Sara Stahl^1^, Walid Makhoul^2^, Yvette Sheline^2^, Monique, Ernst^1^, Christian Grillon^1^

1. Section on Neurobiology of Fear and Anxiety
   National Institute of Mental Health
   National Institutes of Health
   Bethesda, MD, USA
2. Center for Neuromodulation in Depression and Stress
   Department of Psychiatry
   University of Pennsylvania
   Philadelphia, PA, USA

**Corresponding Author:**

Nicholas Balderston

Center for Neuromodulation in Depression and Stress

3700 Hamilton Walk, Richards D302

Philadelphia, PA, 19104

Phone: 215-746-3058

Fax: 215-573-8556

[nicholas.balderston@pennmedicine.upenn.edu](mailto:nicholas.balderston@pennmedicine.upenn.edu)

**Keywords:** generalized anxiety disorder, social anxiety disorder, working memory, threat, dorsolateral prefrontal cortex, functional magnetic resonance imaging

**Abstract**

One of the hallmarks of anxiety disorders is impaired cognitive control, affecting working memory (WM). The dorsolateral prefrontal cortex (dlPFC) is critical for WM, however it is still unclear how dlPFC activity relates to WM impairments in patients. Forty-one healthy volunteers and 32 anxiety (general and/or social anxiety disorder) patients completed the Sternberg WM paradigm during safety and unpredictable shock threat. On each trial a series of letters was presented, followed by brief retention and response intervals. On low and high load trials, subjects retained the series (5 and 8 letters, respectively) in the original order, while on sort trials subjects rearranged the series (5 letters) in alphabetical order. We sampled BOLD activity during retention using a bilateral anatomical dlPFC mask. Compared to controls, patients showed increased reaction time during high load, greater right dlPFC activity, and reduced dlPFC activity during threat. These results suggest that WM performance for patients and controls may rely on distinct patterns of dlPFC activity with patients requiring bilateral dlPFC activity. These results are consistent with reduced efficiency of WM in anxiety patients. This reduced efficiency may be due to an inefficient allocation of dlPFC resources across hemispheres or a decreased overall dlPFC capacity.

**Introduction**

Anxiety disorders are one of the most commonly diagnosed classes of mental disorders, affecting 1 in 5 individuals in a given year (Kessler and Chiu, 2005). One of the hallmarks of these conditions is an inability to focus attention (First *et al.*, 2012), which makes it difficult for anxious participants to perform attentionally demanding tasks (Eysenck *et al.*, 2007), like those that require manipulating items in working memory (WM) (Vytal *et al.*, 2012; Vytal *et al.*, 2013). One critical region thought to be important for WM manipulation is the dorsolateral prefrontal cortex (dlPFC) (Balderston, Vytal, *et al.*, 2016). Using threat of shock to induce anxiety is one approach to studying the relationship between anxiety and cognition (Robinson *et al.*, 2013; Grillon *et al.*, 2019); however, additional research is needed to determine whether this approach can shed light on the cognitive symptoms seen in anxious patients and inform the identification of treatment targets for anxiety disorders (Balderston *et al.*, 2020). Therefore, the purpose of this study is to examine WM-related dlPFC activity in anxiety patients and controls and whether this activity is affected by manipulations in anxiety state.

Although there are few WM studies conducted in anxiety patients, the results often suggest processing deficits in WM-related regions including the dlPFC. For instance, patients with generalized anxiety disorder (GAD) show reduced left dlPFC activation during WM maintenance (Moon and Jeong, 2015; Moon *et al.*, 2016; Moon and Jeong, 2017) but greater activation in WM-related regions when presented with emotional distractors (Moon and Jeong, 2015; Park *et al.*, 2016). GAD patients also show reduced activation in frontal, parietal, and cerebellar regions important for WM maintenance compared to controls during a WM suppression task (Diwadkar *et al.*, 2017). Patients with posttraumatic stress disorder (PTSD) have reduced default mode network (DMN) deactivation during the 3-back task (Landré *et al.*, 2012), and reduced frontoparietal activity during memory updating (Shaw *et al.*, 2009). Patients with anxious major depressive disorder show reduced left prefrontal beta-band desynchronization during the n-back task (Ionescu *et al.*, 2015). Together these results suggest that deficits in WM-related processing may be a dimensional indicator of anxiety, cutting across anxiety disorders.

Despite these deficits in WM-related processing, anxiety patients are often able to perform at a similar level of accuracy as healthy controls, although at the cost of slower response, suggesting the implementation of compensatory mechanisms to maintain performance. A prime candidate region for this compensatory processing is the right dlPFC. For instance, high anxious subjects show elevated right dlPFC activity during WM maintenance compared to low anxious subjects (Basten *et al.*, 2012). Similarly, healthy subjects show elevated right dlPFC activity during complex cognitive tasks performed under threat of shock (Oei *et al.*, 2012; Balderston, Hsiung, *et al.*, 2017; Balderston, Liu, *et al.*, 2017). However, this argument is inconsistent with neuromodulatory data targeting the right dlPFC, showing that excitatory rTMS increases anxiety (Balderston *et al.*, 2020) while inhibitory rTMS reduces anxiety (Chen *et al.*, 2013). In addition, retention interval activity is often left-lateralized, especially when verbal stimuli are used (Altamura *et al.*, 2010; Rottschy *et al.*, 2012), making the functional significance of these anxiety-related right dlPFC changes unclear. The key question then is whether right dlPFC contributes to or detracts from cognitive control in anxious patients and whether this right dlPFC activity could be a dimensional indicator of anxiety, consistent with the NIMH domain criteria (RDoC) initiative (Insel *et al.*, 2010; Insel, 2014).

Accordingly, we recruited a heterogeneous sample of patients meeting criteria for an anxiety disorder, and compared left and right dlPFC activity during retention interval between these patients and controls in periods of safety and threat. We chose to 1) contrast left and right BOLD activity because state, trait, and clinical anxiety have been shown to impact task-related right dlPFC activity (Basten *et al.*, 2011; Balderston, Vytal, *et al.*, 2016; Balderston, Hsiung, *et al.*, 2017), but the Sternberg task is known to preferentially activate the left dlPFC (Altamura *et al.*, 2010; Rottschy *et al.*, 2012), and 2) vary the cognitive load of the task by adding a WM manipulation component (letter sorting), in the hope of revealing cognitive control deficits in the patients, characterized by increased reaction time (Eysenck *et al.*, 2007; Cornwell *et al.*, 2011). Finally, since previous research has shown that state anxiety can negatively impact working memory performance and WM-related dlPFC activity (Vytal *et al.*, 2012; Vytal *et al.*, 2013; Balderston, Vytal, *et al.*, 2016; Vytal *et al.*, 2016), we chose to investigate these relationships during periods of safety and threat. We also expected that these cognitive control deficits would be accompanied by an increase in right dlPFC activity in patients compared to controls. However, given that excitatory neuromodulation to the right dlPFC can be anxiogenic and inhibitory neuromodulation to the right dlPFC can be anxiolytic, it is currently unclear whether the right dlPFC contributes to or detracts from cognitive control. Accordingly, it is currently unclear whether right dlPFC activity should be positively or negatively correlated with performance. A positive correlation would suggest that right dlPFC contributes to cognitive control, maintaining performance at the level of the controls, albeit less efficiently. In contrast, a negative correlation would suggest that the right dlPFC detracts from cognitive control, and contributes to the deficits seen in performance.

**Materials and Methods**

**Participants**

Participants were recruited from the Washington DC metro area via flyers, advertisements, and postings on listservs (See Supplemental Table 1 for demographic information). All participants were screened by a trained clinician who administered a structured clinical interview (First *et al.*, 2012). Subjects also completed the Beck Depression Inventory (Beck *et al.*, 1996) and the Beck Anxiety Inventory (Beck *et al.*, 1988). Subjects were included if they were between 18 and 50 years of age, physically healthy, and able to understand the instructions in English. Subjects were excluded if they had 1) neurological or other physical issues or were taking medications that may have impacted the study, 2) alcohol/substance dependence, met criteria for an Axis 1 psychiatric disorder (healthy subjects), 3) first degree relative with a psychotic disorder, or 4) MRI contraindications. Additionally, patients were included if they met criteria for one or more of the following disorders: generalized anxiety disorder (GAD), social anxiety disorder (SAD), post-traumatic stress disorder (PTSD), panic disorder (PD), or specific phobia (SP). A full list of the inclusion/exclusion criteria can be found at: <https://clinicaltrials.gov/ct2/show/NCT00047853>. We recruited 33 patients (26 female) with an average age of 30.41 ± 7.44. One subject was lost due to scanner failure. Of the remaining patients, 12 had a primary diagnosis of GAD, 7 for SAD, 12 for comorbid GAD/SAD, and 2 for comorbid GAD/SAD/PD. We recruited 46 healthy control subjects (26 female) with an average age of 26.8 ± 4.41. Patients were on average significantly older than controls, so age was included as a covariate where appropriate below (*t*(71) = 2.821; *p* = 0.006). Three control subjects were excluded for motion during the fMRI scans, and 2 withdrew from the study. All subjects gave written informed consent approved by the National Institute of Mental Health (NIMH) Combined Neuroscience Institutional Review Board and were compensated for their time.

**Procedure**

**Sternberg Working Memory Task** (Balderston, Quispe-Escudero, *et al.*, 2016)**.** On each trial, subjects were sequentially presented a series of 5 or 8 letters, followed by a brief retention period (See Figure 1). Prior to the letter series, subjects saw a 1 second fixation that indicated the trial type (low = “maintain 5 letters”, high = “maintain 8 letters”, sort = “sort 5 letters”). On low and high “maintain” trials (low = 5 letters; high = 8 letters), subjects rehearsed the series in order. On “sort” trials (5 letters), subjects rearranged the letters in alphabetical order. Following the retention interval, subjects were presented with a letter and a number, and made a forced choice button press indicating whether the position of the letter in the original (low and high trials) or alphabetical (sort trials) series matched the number.

There were 4 runs, each with 26 trials. Half of the trials were matches, meaning the position of the letter correctly matched the number shown, and half were mismatches. Low, high, and sort trials were randomly shuffled within alternating blocks of safe and threat (2 blocks each per run). The safe and threat blocks were signaled using colored banners with the words “Safe” and “Threat” that were present on the screen for the duration of the block. Two threat trials per run included a shock presentation and were discarded. On each trial, the fixation period lasted 1s, the encoding period lasted between 3 and 5.5 s, the retention period lasted between 3 and 5.5, and the response period lasted 3 s. The duration of the encoding and retention intervals were jittered across trials to allow for deconvolution of the BOLD response to the separate intervals. Even so there remained some collinearity between the regressors (*mean correlation* = 0.42), suggesting that some variability was shared across intervals (*mean r^2^* = 0.177). However, it should be noted that partial correlation coefficients were used to model the BOLD response, so the results do not include this shared variability. The total trial length was 20 seconds, and the intertrial interval varied based on the duration of the encoding and retention intervals. After each run, subjects were asked to rate their anxiety during the safe and threat periods on a scale from 1 (not anxious) to 10 (extremely anxious; See Supplemental Table 1).

**Shock.** The shock stimulus was a 200 Hz train of stimulation delivered for 100 ms to the right wrist using a constant current stimulator (Digitimer #DS7A, Ft. Lauderdale, FL). Two 11 mm disposable Ag/AgCl electrodes (Biopac Item number EL508; Goleta, CA), spaced ~2 cm apart delivered the shock. Intensity was determined at the start of the experiment using an individualized thresholding procedure. Subjects rated each shock on a scale from 1 (not uncomfortable) to 10 (uncomfortable but tolerable), and shocks were delivered throughout the experiment at the level that subjects rated as their level 10 (See Supplemental Table 1). Subjects also rated the shock after each run on the same scale, and values did not substantially change in these post-run ratings (See Supplemental Table 1).

**Scans.** Scanning took place in a Siemens 3T Skyra MRI scanner with a 32-channel head coil. Subjects viewed the task through a coil mounted mirror system. We acquired a T1-weighted MPRAGE (TR = 2530 ms; TE1 = 1.69 ms; TE2 = 3.55 ms; TE3 = 5.41 ms; TE4 = 7.27 ms; flip angle = 7°) with 176, 1 mm axial slices (matrix = 256 mm × 256 mm; field of view (FOV) = 204.8 mm × 204.8 mm). During the task, we acquired whole-brain multi-echo echoplanar images (EPI; TR = 2000 ms; TEs = 13.8, 31.2, 48.6 ms; flip angle = 70°) comprised of 32, 3 mm axial slices (matrix = 64 mm × 64 mm; FOV = 192 mm × 192 mm) aligned to the AC-PC line. In addition, we acquired a reverse phase-encoded “blip” EPI image to correct for geometric distortion in the EPI data.

**Performance analysis.** For both accuracy and reaction time, we performed a 2 (group: patient vs. control) by 2 (condition: safe vs. threat) by 3 (load: low vs. high vs. sort) mixed model ANOVA. We then characterized the interactions using post hoc t-tests.

**fMRI Pre-processing.** Preprocessing was done using afni_proc.py (Kundu *et al.*, 2012), which included slice-timing correction, despiking, volume registration, identification of non-BOLD components using a TE-dependent independent components analysis (ICA) (Kundu *et al.*, 2012), scaling, EPI distortion correction, nonlinear normalization to the MNI template, and blurring with a 6 mm FWHM gaussian kernel. Timeseries were then scrubbed for motion (threshold set at > .5 mm RMS), and modeled using a first level GLM that included the following regressors of no interest: baseline (polynomial estimates), 6 motion parameters and their derivatives, the non-BOLD component timeseries, shock onsets, and button presses. The GLM also included regressors of interest corresponding to the encoding, retention, and response interval of the Sternberg trials. These intervals were modeled as variable duration blocks independently for the different conditions (i.e. safe vs. threat, low vs. high vs. sort).

**fMRI analysis.** The resulting beta maps from the first level GLM were then analyzed using an *a priori* region of interest (ROI) approach focused on the left and right dlPFC, and an exploratory whole-brain voxelwise approach. For the ROI analysis, we used neurosynth (Yarkoni *et al.*, 2011) to generate a bilateral mask of the dlPFC by searching the term “dlPFC”, saving the resulting association test map, and extracting the 2 primary clusters corresponding to the left and right dlPFC. We chose the anatomical search term “dlPFC”, as opposed to a functional search term like “working memory” for two reasons. First, we wanted the selection of the ROI to be independent of the factors in the experiment. Second, we were primarily interested in dlPFC activity, specifically because it is a common therapeutic target for neuromodulation (O’Reardon *et al.*, 2007). We then averaged the beta values for the retention interval across voxels within each dlPFC ROI and performed a 2 (group: patient vs. control) by 2 (condition: safe vs. threat) by 3 (load: low vs. high vs. sort) x 2 (hemisphere: left vs. right) mixed model ANOVA on the values. Note that we chose an ROI approach for the focus of this paper because we wanted to characterize the activity in the dlPFC across all experimental conditions in the current study. Given the large number of factors in this study, the design contains a large number of degrees of freedom, and thus a large number of distinct voxelwise maps in the omnibus analysis. Accordingly, it can be difficult to interpret the results from main effects and higher order interactions in overlapping but non-identical regions.

In addition to the *a priori* ROI analysis of the dlPFC, we also conducted exploratory voxelwise analyses at the whole brain level. As before, we extracted the betas from the first level GLM corresponding to the encoding, retention, and response intervals and then performed three 2 (group: patient vs. control) by 2 (condition: safe vs. threat) by 3 (load: low vs. high vs. sort) mixed model ANOVAs on the values for each interval. We used cluster thresholding based on 10,000 Monte Carlo simulations to correct for multiple comparisons (Forman *et al.*, 1995). We chose a 2-tailed voxelwise p-value of 0.001, used a non-Gaussian autocorrelation function that better approximates BOLD data to estimate the smoothness of the residuals (Cox *et al.*, 2017), and clustered voxels with adjoining faces and edges. The result was a minimum cluster size of 41, 3 mm isotropic voxels. Interactions were decomposed using post hoc t-tests, and the results are reported in Supplemental Tables 2-4.

**Results**

**Accuracy**

For accuracy, we performed a group by condition by load mixed model ANOVA (See Table 1 and Figure 2A). There was a significant main effect of load (*F*(2,142) = 18.419; *p* < 0.001; *Partial eta^2^* = 0.21), but no other main effects or interactions (*p*s > 0.05). To characterize this main effect, we performed paired sample t-tests for each of the 3 possible load comparisons and found that accuracy decreased significantly from low to sort to high (low > sort: *t*(72) = 3.074; *p* = 0.003; *d* = 0.36; sort > high: *t*(72) = 3.03; *p* = 0.003; *d* = 0.35; low > high: *t*(72) = -6.736; *p* < 0.001; *d* = 0.79).

**Reaction Time**

For reaction time, we performed a group by condition by load mixed model ANOVA (See Table 1 and Figure 2B). As with accuracy, we found a significant main effect for load (*F(*2,142) = 30.557; *p* < 0.001; *Partial eta^2^* = 0.3). In addition, we found a significant load by group interaction (*F(*2,142) = 3.489; *p* = 0.033; *Partial eta^2^* = 0.05). However, there were no other significant main effects or interactions (*p*s > 0.05). With age as a covariate, the load by group interaction is no longer significant (*F(*2,142) = 2.577; *p* = 0.08; *Partial eta^2^* = 0.01).

To characterize the load main effect, we performed paired sample t-tests for each of the 3 possible load comparisons and found that RT increased significantly from low to sort to high (low < sort: *t*(72) = 3.672; *p* < 0.001; *d* = 0.43; sort < high: *t*(72) = -3.236; *d* = 0.38; *p* = 0.002; low < high: *t*(72) = 9.113; *p* < 0.001; *d* = 1.07). To characterize the load by group interaction, we performed t-tests comparing RT for controls vs. patients for each level of load. We found that patients were significantly slower than controls for high trials (*t*(71) = 2.159; *p* = 0.034; *d* = 0.51), but not low (*t*(71) = 0.718; *p* = 0.475; *d* = 0.17) or sort (*t*(71) = 1.72; *p* = 0.09; *d* = 0.4) trials.

**dlPFC BOLD**

To examine retention interval activity in the dlPFC, we averaged the beta values within ROIs for the left and right dlPFC (See Table 2 and Figure 1C) and performed a group by condition by load by hemisphere mixed model ANOVA on the values. We found significant effects for the following factors: load (*F*(2,142) = 21.587; *p* < 0.001; *Partial eta^2^* = 0.23), hemisphere (*F*(1,71) = 11.47; *p* = 0.001; *Partial eta^2^* = 0.14), condition by load (*F*(2,142) = 3.075; *p* = 0.049; *Partial eta^2^* = 0.04), load by hemisphere (*F*(2,142) = 15.285; *p* < 0.001; *Partial eta^2^* = 0.18), condition by group (*F*(1,71) = 7.359; *p* = 0.008; *Partial eta^2^* = 0.09), and hemisphere by group (*F*(1,71) = 4.011; *p* = 0.049; *Partial eta^2^* = 0.05). However, it should be noted (as shown below) that the main effect of hemisphere is driven primarily by the control group. Group interaction results are similar if age is included as a covariate.

To characterize these effects, we plotted the corresponding comparisons in Figure 3 and conducted post hoc t-tests to examine the directionality of the effects. First, for the main effect of load (Figure 3A), we observed an increase in dlPFC BOLD from low to high (*t*(72) = 3.36; *p* = 0.001; *d* = 0.4) and high to sort (*t*(72) = -3.577; *p* = 0.001; *d* = -0.42). Note that the sort > low comparison is also significant (*t*(72) = 6.234; *p* < 0.001; *d* = 0.72). For the hemisphere main effect, we observed significantly more left compared to right dlPFC activity (See Figure 3B and F-test above). For the condition by load interaction, we observed significantly less dlPFC activity during threat compared to safe for the sort condition (*t*(72) = 2.877; *p* = 0.005; *d* = 0.33) but not the low (*t*(72) = 0.003; *p* = 0.998; *d* = 0) or high (*t*(72) = -0.019; *p* = 0.985; *d* = 0) condition (See Figure 3C). For the load by hemisphere interaction, we observed significantly more left compared to right dlPFC activity for the low (*t*(72) = 5.271; *p* < 0.001; *d* = 0.61) and sort (*t*(72) = 4.271; *p* < 0.001; *d* = 0.5) conditions, but not the high (*t*(72) = 0.111; *p* = 0.912; *d* = 0.01) condition (See Figure 3D). For the group by condition interaction, we observed significantly less dlPFC activity during threat compared to safe for the patients (*t*(31) = 2.635; *p* = 0.013; *d* = 0.46) but not the control (*t*(40) = -0.833; *p* = 0.41; *d* = -0.13) subjects (See Figure 3E). Finally, for the group by hemisphere interaction, we observed significantly less activity in the right compared to left dlPFC for the controls (*t*(40) = 3.957; *p* < 0.001; *d* = 0.62) but not the patients (*t*(31) = 0.96; *p* = 0.344; *d* = 0.17; See Figure 3F).

Importantly, these results seem to be specific to working memory maintenance. When we run the same group by condition by load by hemisphere mixed model ANOVA on the values extracted for the encoding and response windows, we see no significant main effects or interactions for group or condition (*p*s > 0.05).

To understand whether this right dlPFC activation contributes to or detracts from performance, we correlated right dlPFC activity during the sort condition with accuracy (See Figure 4). Consistent with the idea that the right dlPFC contributes to cognitive control, especially in patients, we found a significant positive correlation between right dlPFC activity and accuracy for patients (*r*(31) = .38; *p* = 0.032) and a trend-level positive correlation in controls (*r*(40) = .25; *p* = 0.115). Trend level correlations were also seen for the left dlPFC for both patients (*r*(31) = .26; *p* = 0.151) and controls (*r*(40) = .25; *p* = 0.115).

**Exploratory whole-brain BOLD**

The two main goals of the whole-brain voxelwise analysis were to 1) dissociate BOLD activity to the encoding, retention, and response intervals, and 2) dissociate BOLD activity associated with WM maintenance and manipulation. Accordingly, we modeled the encoding, retention, and response intervals separately with variable duration blocks and conducted 3 separate group by condition by load mixed model ANOVAs corresponding to the betas for each interval (See Tables 2-4). To characterize the load manipulation, we also conducted two orthogonal planned comparisons for each interval that captured responses related to task difficulty (low vs. high and sort; Figure 5A, C, E) and working memory manipulation (high vs. sort; Figure 5B, D, F). As can be seen from Figure 5, we observed distinct patterns of BOLD activity for the two comparisons across the encoding, retention, and response intervals. Importantly, we observed the most robust dlPFC activity during the retention interval for both comparisons, consistent with the ROI analysis.

Aside from the load main effects, there were several other patterns worth noting. Threat tended to decrease activity in several midline regions including the cuneus, the middle cingulate and the medial prefrontal cortex during the encoding period for sort, while decrease the activity during the high condition. Main effects for condition were largely located in task positive parietal and occipital regions, replicating previous work, and tended to occur during the processing of external stimuli (encoding and response intervals) rather than the retention interval (Larson *et al.*, 2009; Hoffmann *et al.*, 2012; Hu *et al.*, 2013; Torrisi *et al.*, 2016; Balderston, Hsiung, *et al.*, 2017; Balderston, Hale, *et al.*, 2017). Together, these results support the use of the Sternberg task as a method for isolating responses to unique WM processes, and suggest that threat interactions with WM may be most robust during encoding and retrieval of items in WM.

**Discussion**

Little is known about mechanisms underlying WM impairment in anxiety patients as there is a dearth of investigation on this topic (Moon *et al.*, 2016; Park *et al.*, 2016). In this study we used the Sternberg WM paradigm to assess retention interval activity in anxiety patients and healthy controls during periods of safety and shock threat. Our 2 primary findings were that 1) anxiety patients were slower than controls when task demands were high, and 2) anxiety patients recruited bilateral dlPFC activation, while controls were able to complete the task using primarily the left dlPFC. These results are consistent with the attentional control theory, which posits that anxiety patients can overcome attention control deficits and perform at similar levels as controls because of compensatory neural processes (Eysenck *et al.*, 2007). Interestingly, right dlPFC BOLD activity was positively correlated with accuracy during the sort condition for patients but not controls. This is puzzling, given that excitatory neuromodulation of the right dlPFC has been shown to be anxiogenic (Balderston *et al.*, 2020), while inhibitory neuromodulation of the right dlPFC has been shown to be anxiolytic (Chen *et al.*, 2013).

While it would be tempting to conclude that anxiety patients suffer from a deficiency in left dlPFC processing, which is overcome by recruitment of the right dlPFC, our current results alone are not sufficient to support this conclusion. For this, we would need to show significantly less left and significantly more right dlPFC activity in the patients compared to the controls. Unfortunately, our current study is not sufficiently powered to demonstrate these effects. Even so, support for this left hemisphere deficit hypothesis comes from EEG data showing hypoactivation in the left hemisphere for both anxiety and depression, which is frequently comorbid with anxiety (Davidson *et al.*, 1990; Davidson, 2004). Importantly, these data form the basis of the therapeutic application of transcranial magnetic stimulation (TMS) in individuals with depression (O’Reardon *et al.*, 2007; Chen *et al.*, 2013). The most common application of TMS for depression is to boost left dlPFC activity through left-lateralized excitatory stimulation (i.e. high frequency or intermittent theta burst stimulation) (O’Reardon *et al.*, 2007). Interestingly, our current results may also provide insight into the mechanism of action of the anxiolytic effects of inhibitory rTMS to the right dlPFC (Chen *et al.*, 2013). If indeed right dlPFC recruitment is a compensatory mechanism for diminished capacity in the left dlPFC, perhaps inhibiting right dlPFC activity forces the left dlPFC to work harder leading to plastic changes and improved functioning. If this is the case, one should expect a gradual shift in dlPFC activity over the course of right-lateralized inhibitory rTMS dlPFC treatment, normalizing the pattern of dlPFC during the retention interval for these patients. However, it should be noted that inhibitory rTMS to the right dlPFC may impair WM performance in anxiety patients, a possibility that should be considered in future trials. Future work should test this hypothesis. Although not included in the current sample, PTSD patients have been shown to benefit from both excitatory (Boggio *et al.*, 2010) and inhibitory (Kozel *et al.*, 2018) right dlPFC stimulation, suggesting that additional work is needed to show the generality of the above hypothesis.

Behaviorally, we observed increased reaction times in patients compared to controls for the high load condition, which was also the most difficult. However it should be noted that after controlling for age, this effect is only a trend. Like the dlPFC results, these behavioral results are consistent with the attentional control theory (Eysenck *et al.*, 2007), suggesting that high anxious individuals process information less efficiently, requiring more time to perform at the same level of accuracy (Lee, 1999; Richards *et al.*, 2000). Again, it would be tempting to attribute this slower reaction time to the bilateral dlPFC effects that we observed in the patients. However, the increased reaction time in the patients was greatest for the high load condition, while dlPFC activity was strongest for the sort condition. While this data alone should not rule out a dlPFC/RT connection, it should also be noted that there were no significant correlations between reaction time and dlPFC activity in the patients or the controls. Anecdotally it often seems as if the anxiety patients have a stronger desire to do well on the task, which can lead to more effort on difficult trials. Although we did not formally test this hypothesis in the current work, there is experimental evidence that cognitive performance anxiety can lead to increases in reaction time during working memory tasks (Angelidis *et al.*, 2019).

In addition to slower reaction times and less efficient dlPFC processing, it is also known that depression and anxiety patients have trouble filtering distractors from WM memory (Meconi *et al.*, 2013; Qi *et al.*, 2013; Stout *et al.*, 2013; Stout *et al.*, 2015; Park *et al.*, 2016; Stout *et al.*, 2017). In the current study, patients showed reduced dlPFC activity during threat, which may reflect arousal-related impairments in top-down control that could affect distractor suppression (Stout *et al.*, 2018). This filtering difficulty has been repeatedly shown using spatial WM tasks that rely on contralateral delay activity (CDA), an event-related potential measured in parietal regions using electroencephalography (EEG) (Vogel and Machizawa, 2004). In a typical task, subjects must study and retain the spatial configuration of items in one visual hemifield over the other. Importantly, EEG signal power in the contralateral hemispheres is linearly related to the number of attended items in the visual display (Vogel and Machizawa, 2004). Importantly, when distractors are also presented in the visual display, highly anxious individuals show contralateral delay activity (CDA) responses that scale not only with the number of items to be attended, but also with the number of items to be ignored (Qi *et al.*, 2013; Stout *et al.*, 2013; Stout *et al.*, 2015). This effect is especially pronounced when the items carry a negative emotional valence (Meconi *et al.*, 2013; Park *et al.*, 2016; Stout *et al.*, 2017). Similarly, highly anxious individuals have difficulty task switching (Gustavson *et al.*, 2017) and updating items in WM (Gustavson and Miyake, 2016), which could be interpreted as an inability to suppress previously task-relevant data similar to the deficits seen with task irrelevant distractors. It is currently unclear how this failure to filter distractors relates to our current findings. However, there is structural connectivity evidence to suggest a potential link. A recent study looked at the relationship between frontoparietal connectivity and WM capacity and found that those with a strong connectivity had higher WM capacity than those with weak connectivity (Ekman *et al.*, 2016). One possible explanation is that the deficient filtering is again driven by diminished left dlPFC capacity and/or diminished left dlPFC/parietal connectivity. It may be possible to test this hypothesis by administering a course of therapeutic rTMS to the left or right dlPFC and measuring CDA activity as a function of both target and distractor load.

In addition to the group effects, we also observed threat-related reductions in dlPFC activity during sort trials. Notably, previous research with the Sternberg task has shown that the sort condition engages working memory manipulation processes that drive greater dlPFC activity (Altamura *et al.*, 2007), an effect that we replicate here (i.e. sort > high in dlPFC). This suggests that threat impairs working memory manipulation processes in the dlPFC. Indeed, there have been a number of studies investigating the effect of threat on WM, primarily using the n-back WM task (Vytal *et al.*, 2012; Clarke and Johnstone, 2013; Vytal *et al.*, 2013; Patel *et al.*, 2015; Ernst *et al.*, 2016). The major findings from these studies are that 1) threat reduces accuracy, especially on low load WM trials (Vytal *et al.*, 2012; Vytal *et al.*, 2013; Patel *et al.*, 2015), and 2) threat reduces overall dlPFC processing (Clarke and Johnstone, 2013). Although, we did not observe an effect of threat on accuracy in the current work, these results replicate our previous work using the Sternberg WM task during threat (Balderston, Quispe-Escudero, *et al.*, 2016), suggesting that either the dlPFC effects shown here are below the threshold needed to cause a behavioral effect, or that the behavioral effects observed with the n-back task may be due to some other aspects of the task. Given that the n-back task features constant encoding, maintenance, and retrieval, it is difficult to relate performance to neural activity during distinct phases of the task. Future work using adaptations of the n-back suited for event related fMRI analyses might provide a better understanding of the mechanisms mediating threat-related performance deficits on this task (Fales *et al.*, 2008).

**Strengths and Limitations**

The primary strengths of this study are that we directly compared dlPFC BOLD in a relatively large (for fMRI) sample of *unmedicated* anxiety patients and healthy control subjects. Another strength of this study is that we used a version of the Sternberg WM paradigm that was optimized for fMRI, allowing us to dissociate encoding, retention, and retrieval processes and show retention-specific differences across groups.

The main limitation of the current work is that we have a heterogeneous group of anxiety patients, most of them comorbid and meeting criteria for GAD, SAD, and PD with some degree of comorbidity. Although it is beyond the scope of the current work to distinguish between these anxiety subpopulations, our results are similar if we look at those that meet either the criteria for GAD or SAD. Future studies should be conducted with adequate samples of patients meeting criteria for each of these disorders to identify any disorder-specific differences in retention-interval BOLD responses.

Another limitation is that our sample size, albeit large, may not have been sufficient to detect significant correlations between accuracy and left dlPFC activity in the patients or controls. Accordingly, it would be preliminary (and largely inconsistent with the literature) to suggest that left dlPFC activity does not contribute verbal working memory. However, we were able to detect a significant correlation between right dlPFC activity and accuracy in the anxiety patients, suggesting that activity in this region is particularly important for these individuals. In other words, while previous research suggests that the left dlPFC may be necessary for successful working memory performance generally (Altamura *et al.*, 2010; Rottschy *et al.*, 2012), our research provides indirect correlational (i.e. fMRI) evidence that left dlPFC activity may not be sufficient for successful working memory performance in anxiety patients.

Additionally, although we jittered the onsets of the maintenance and retrieval periods, there were still correlations between the regressors. This is impossible to avoid because the encoding, maintenance, and retrieval periods must be presented in order for the task to function correctly. However, it is important to note that the data reported from the manuscript come from the partial correlations between the regressors and the BOLD timeseries, so the results do not include this shared variability. This means that even though we are reducing the sensitivity of our analysis, we still maintain specificity.

One final limitation is that we did not objectively measure the anxiety level (e.g. through startle potentiation) of our subjects to assess the effectiveness of the threat manipulation. However, it should be noted that threat of unpredictable shock has been repeatedly used in our lab and others as a robust anxiogenic experimental condition (Charney *et al.*, 1984; Lang *et al.*, 1990; Grillon *et al.*, 1994; Morgan *et al.*, 1995; Böcker *et al.*, 2004; Grillon, 2008; Balderston *et al.*, 2015; Balderston, Quispe-Escudero, *et al.*, 2016)**.**

**Conclusions**

The current work suggests that anxiety patients process items in WM differently than healthy controls, which may prove to be a key avenue for future treatments for these individuals. Future studies using noninvasive neuromodulation may be able to correct this bilateral retention interval processing by either boosting left dlPFC excitability or attenuating right dlPFC excitability, an approach that has already been shown to be effective in treatments for major depressive disorder (O’Reardon *et al.*, 2007; Dell’Osso *et al.*, 2015).

**Author contributions:** The study was designed by NLB, CG, ME. The data were collected by NLB, EF, AH, JL, AT, SS. The analysis was conducted by NLB. The manuscript was prepared by NLB, WM, YS, ME, CG.

**Acknowledgments:** This study utilized the high-performance computational capabilities of the Biowulf Linux cluster at the National Institutes of Health, Bethesda, MD. (<https://hpc.nih.gov/>). The authors all work at the National Institutes of Health. The views expressed here are the authors own and do not necessarily reflect the views of the NIH, DHHS, or the US Federal Government.

**Financial Support:** Financial support of this study was provided by the Intramural Research Program of the National Institute of Mental Health, ZIAMH002798 (ClinicalTrial.gov Identifier: NCT00047853: Protocol ID 02-M-0321).

**Conflicts of Interest:** The authors report no biomedical financial interests or potential conflicts of interest.

**Ethical Standards:** The authors assert that all procedures contributing to this work comply with the ethical standards of the relevant national and institutional committees on human experimentation and with the Helsinki Declaration of 1975, as revised in 2008.

**References**

Altamura, M., Elvevåg, B., Blasi, G., et al. (2007). Dissociating the effects of Sternberg working memory demands in prefrontal cortex. *Psychiatry research*, **154**, 103–14

Altamura, M., Goldberg, T.E., Elvevåg, B., et al. (2010). Prefrontal Cortex Modulation during Anticipation of Working Memory Demands as Revealed by Magnetoencephalography. *International journal of biomedical imaging*, **2010**

Angelidis, A., Solis, E., Lautenbach, F., et al. (2019). I’m going to fail! Acute cognitive performance anxiety increases threat-interference and impairs WM performance. *PLoS ONE*, **14**, 1–25

Balderston, N.L., Beydler, E.M., Roberts, C., et al. (2020). Mechanistic link between right prefrontal cortical activity and anxious arousal revealed using transcranial magnetic stimulation in healthy subjects. *Neuropsychopharmacology*, **45**, 694–702

Balderston, N.L., Hale, E., Hsiung, A., et al. (2017). Threat of shock increases excitability and connectivity of the intraparietal sulcus. *eLife*, **6**

Balderston, N.L., Hsiung, A., Ernst, M., et al. (2017). Effect of threat on right dlPFC activity during behavioral pattern separation. *Journal of Neuroscience*, **37**

Balderston, N.L., Liu, J., Roberson-Nay, R., et al. (2017). The relationship between dlPFC activity during unpredictable threat and CO<inf>2</inf>-induced panic symptoms. *Translational Psychiatry*, **7**

Balderston, N.L., Mathur, A., Adu-Brimpong, J., et al. (2015). Effect of anxiety on behavioural pattern separation in humans. *Cognition and Emotion*, **9931**, 1–11

Balderston, N.L., Quispe-Escudero, D., Hale, E., et al. (2016). Working memory maintenance is sufficient to reduce state anxiety. *Psychophysiology*

Balderston, N.L., Vytal, K.E., O’Connell, K., et al. (2016). Anxiety Patients Show Reduced Working Memory Related Dlpfc Activation During Safety and Threat. *Depression and Anxiety*, **12**, 1–12

Basten, U., Stelzel, C., Fiebach, C.J. (2012). Trait anxiety and the neural efficiency of manipulation in working memory. *Cognitive, affective & behavioral neuroscience*, **12**, 571–88

Basten, U., Stelzel, C., Fiebach, C.J. (2011). Trait anxiety modulates the neural efficiency of inhibitory control. *Journal of cognitive neuroscience*, **23**, 3132–45

Beck, A.T., Brown, G.K., Steer, R.A. (1996). BDI-II Manual. *Journal of health psychology*, **17**

Beck, A.T., Epstein, N., Brown, G., et al. (1988). An inventory for measuring clinical anxiety: psychometric properties. *Journal of consulting and clinical psychology*, **56**, 893–97

Böcker, K.B.E., Baas, J.M.P., Leon Kenemans, J., et al. (2004). Differences in startle modulation during instructed threat and selective attention. *Biological Psychology*, **67**, 343–58

Boggio, P.S., Rocha, M., Oliveira, M.O., et al. (2010). Noninvasive brain stimulation with high-frequency and low-intensity repetitive transcranial magnetic stimulation treatment for posttraumatic stress disorder. *Journal of Clinical Psychiatry*, **71**, 992–99

Charney, D.S., Heninger, G.R., Breier, A. (1984). Noradrenergic function in panic anxiety. Effects of yohimbine in healthy subjects and patients with agoraphobia and panic disorder. *Arch Gen Psychiatry.*, **41**, 751--63.

Chen, J., Zhou, C., Wu, B., et al. (2013). Left versus right repetitive transcranial magnetic stimulation in treating major depression: A meta-analysis of randomised controlled trials. *Psychiatry Research*, **210**, 1260–64

Clarke, R., Johnstone, T. (2013). Prefrontal inhibition of threat processing reduces working memory interference. *Frontiers in human neuroscience*, **7**, 228

Cornwell, B.R., Alvarez, R.P., Lissek, S., et al. (2011). Anxiety overrides the blocking effects of high perceptual load on amygdala reactivity to threat-related distractors. *Neuropsychologia*, **49**, 1363–68

Cox, R.W., Chen, G., Glen, D.R., et al. (2017). FMRI Clustering in AFNI: False-Positive Rates Redux. *Brain Connectivity*, **7**, 152–71

Davidson, R.J. (2004). What does the prefrontal cortex ‘do’ in affect: Perspectives on frontal EEG asymmetry research. *Biological Psychology*, **67**, 219–33

Davidson, R.J., Chapman, J.P., Chapman, L.J., et al. (1990). Asymmetrical brain electrical activity discriminates between psychometrically-matched verbal and spatial cognitive tasks. *Psychophysiology*, **27**, 528–43

Dell’Osso, B., Oldani, L., Camuri, G., et al. (2015). Augmentative repetitive Transcranial Magnetic Stimulation (rTMS) in the acute treatment of poor responder depressed patients: A comparison study between high and low frequency stimulation. *European Psychiatry*, **30**, 271–76

Diwadkar, V.A., Re, M., Cecchetto, F., et al. (2017). Attempts at memory control induce dysfunctional brain activation profiles in Generalized Anxiety Disorder: An exploratory fMRI study. *Psychiatry Research - Neuroimaging*, **266**, 42–52

Ekman, M., Fiebach, C.J., Melzer, C., et al. (2016). Different roles of direct and indirect frontoparietal pathways for individual working memory capacity. *Journal of Neuroscience*, **36**, 2894–2903

Ernst, M., Lago, T., Davis, A., et al. (2016). The effects of methylphenidate and propranolol on the interplay between induced-anxiety and working memory. *Psychopharmacology*, **233**, 3565–74

Eysenck, M.W., Derakshan, N., Santos, R., et al. (2007). Anxiety and cognitive performance: attentional control theory. *Emotion (Washington, D.C.)*, **7**, 336–53

Fales, C.L., Barch, D.M., Burgess, G.C., et al. (2008). Anxiety and cognitive efficiency: differential modulation of transient and sustained neural activity during a working memory task. *Cognitive, affective & behavioral neuroscience*, **8**, 239–53

First, M.B., Spitzer, R.L., Gibbon, M., et al. (2012). *Structured Clinical Interview for DSM-IV®Axis I Disorders (SCID-I), Clinician Version, Administration Booklet*. American Psychiatric Publishing.

Forman, S.D., Cohen, J.D., Fitzgerald, M., et al. (1995). Improved assessment of significant activation in functional magnetic resonance imaging (fMRI): use of a cluster-size threshold. *Magnetic Resonance in Medicine*, **33**, 636–47

Grillon, C. (2008). Greater sustained anxiety but not phasic fear in women compared to men. *Emotion (Washington, D.C.)*, **8**, 410–13

Grillon, C., Ameli, R., Goddard, A., et al. (1994). Baseline and fear-potentiated startle in panic disorder patients. *Biological Psychiatry*, **35**, 431–39

Grillon, C., Robinson, O.J., Cornwell, B., et al. (2019). Modeling anxiety in healthy humans: a key intermediate bridge between basic and clinical sciences. *Neuropsychopharmacology*

Gustavson, D.E., Altamirano, L.J., Johnson, D.P., et al. (2017). Set shifting really impaired in trait anxiety? Only when switching away from an effortfully established task set. *Emotion*, **17**, 88–101

Gustavson, D.E., Miyake, A. (2016). Trait worry is associated with difficulties in working memory updating. *Cognition and Emotion*, **30**, 1289–1303

Hoffmann, M., Lipka, J., Mothes-Lasch, M., et al. (2012). Awareness modulates responses of the amygdala and the visual cortex to highly arousing visual threat. *NeuroImage*, **62**, 1439–44

Hu, K., Padmala, S., Pessoa, L. (2013). Interactions between reward and threat during visual processing. *Neuropsychologia*, **51**, 1763–72

Insel, T., Cuthbert, B.N., Garvey, M., et al. (2010). Research Domain Criteria (RDoC): Toward a new classification framework for research on mental disorders. *American Journal of Psychiatry*, **167**, 748–51

Insel, T.R. (2014). The NIMH research domain criteria (RDoC) project: precision medicine for psychiatry. *American Journal of Psychiatry*, 395–97

Ionescu, D.F., Nugent, A.C., Luckenbaugh, D. a., et al. (2015). Baseline working memory activation deficits in dimensional anxious depression as detected by magnetoencephalography. *Acta Neuropsychiatrica*, 1–10

Kessler, R.C., Chiu, W.T. (2005). Prevalence, severity, and comorbidity of 12-month DSM-IV disorders in the National Comorbidity Survey Replication. *Archives of general …*, **62**, 617–27

Kozel, F.A., Motes, M.A., Didehbani, N., et al. (2018). Repetitive TMS to augment cognitive processing therapy in combat veterans of recent conflicts with PTSD: A randomized clinical trial. *Journal of Affective Disorders*, **229**, 506–14

Kundu, P., Inati, S.J., Evans, J.W., et al. (2012). Differentiating BOLD and non-BOLD signals in fMRI time series using multi-echo EPI. *NeuroImage*, **60**, 1759–70

Landré, L., Destrieux, C., Andersson, F., et al. (2012). Working memory processing of traumatic material in women with posttraumatic stress disorder. *Journal of Psychiatry and Neuroscience*, **37**, 87–94

Lang, P.J., Bradley, M.M., Cuthbert, B.N. (1990). Emotion, attention, and the startle reflex. *Psychological review*, **97**, 377–95

Larson, C.L., Aronoff, J., Sarinopoulos, I.C., et al. (2009). Recognizing threat: a simple geometric shape activates neural circuitry for threat detection. *Journal of cognitive neuroscience*, **21**, 1523–35

Lee, J.H. (1999). Test anxiety and working memory. *Journal of Experimental Education*, **67**, 218–40

Meconi, F., Luria, R., Sessa, P. (2013). Individual differences in anxiety predict neural measures of visual working memory for untrustworthy faces. *Social Cognitive and Affective Neuroscience*, **9**, 1872–79

Moon, C.M., Jeong, G.W. (2017). Functional and morphological alterations associated with working memory dysfunction in patients with generalized anxiety disorder. *Acta Radiologica*, **58**, 344–52

Moon, C.M., Jeong, G.W. (2015). Functional neuroanatomy on the working memory under emotional distraction in patients with generalized anxiety disorder. *Psychiatry and Clinical Neurosciences*, **69**, 609–19

Moon, C.M., Sundaram, T., Choi, N.G., et al. (2016). Working memory dysfunction associated with brain functional deficits and cellular metabolic changes in patients with generalized anxiety disorder. *Psychiatry Research - Neuroimaging*, **254**, 137–44

Morgan, C. a, Grillon, C., Southwick, S.M., et al. (1995). Fear-potentiated startle in posttraumatic stress disorder. *Biological psychiatry*, **38**, 378–85

O’Reardon, J.P., Solvason, H.B., Janicak, P.G., et al. (2007). Efficacy and Safety of Transcranial Magnetic Stimulation in the Acute Treatment of Major Depression: A Multisite Randomized Controlled Trial. *Biological Psychiatry*, **62**, 1208–16

Oei, N.Y.L., Veer, I.M., Wolf, O.T., et al. (2012). Stress shifts brain activation towards ventral ‘affective’ areas during emotional distraction. *Social Cognitive and Affective Neuroscience*, **7**, 403–12

Park, J., Kim, G., Jeong, G., et al. (2016). Brain Activation Patterns Associated with the Effects of Emotional Distracters during Working Memory Maintenance in Patients with Generalized Anxiety Disorder. *Psychiatry Investigation*, **13**, 152

Patel, N., Vytal, K.E., Pavletic, N., et al. (2015). Interaction of threat and verbal working memory in adolescents. *Psychophysiology*, **00**, n/a--n/a

Qi, S., Ding, C., Li, H. (2013). Neural correlates of inefficient filtering of emotionally neutral distractors from working memory in trait anxiety. *Cognitive, affective & behavioral neuroscience*

Richards, A., French, C.C., Keogh, E., et al. (2000). Test-anxiety, inferential reasoning and working memory load. *Anxiety, Stress and Coping*, **13**, 87–109

Robinson, O., Vytal, K.E., Cornwell, B.R., et al. (2013). The impact of anxiety upon cognition: perspectives from human threat of shock studies. *Frontiers in human neuroscience*, **7**, 203

Rottschy, C., Langner, R., Dogan, I., et al. (2012). Modelling neural correlates of working memory: A coordinate-based meta-analysis. *Neuroimage*, **60**, 830–46

Shaw, M.E., Moores, K.A., Clark, R.C., et al. (2009). Functional connectivity reveals inefficient working memory systems in post-traumatic stress disorder. *Psychiatry Research - Neuroimaging*, **172**, 235–41

Stout, D.M., Bomyea, J., Risbrough, V.B., et al. (2018). Aversive Distractors Modulate Affective Working Memory in Frontoparietal Regions. *Emotion*

Stout, D.M., Shackman, A.J., Johnson, J.S., et al. (2015). Worry is associated with impaired gating of threat from working memory. *Emotion*, **15**, 6–11

Stout, D.M., Shackman, A.J., Larson, C.L. (2013). Failure to filter: anxious individuals show inefficient gating of threat from working memory. *Frontiers in human neuroscience*, **7**, 58

Stout, D.M., Shackman, A.J., Pedersen, W.S., et al. (2017). Neural circuitry governing anxious individuals’ mis-allocation of working memory to threat. *Scientific Reports*, **7**, 1–11

Torrisi, S., Robinson, O., O’Connell, K., et al. (2016). The neural basis of improved cognitive performance by threat of shock. *Social Cognitive and Affective Neuroscience*, **11**

Vogel, E.K., Machizawa, M.G. (2004). Neural activity predicts individual differences in visual working memory capacity. *Nature*, **428**, 748–51

Vytal, K.E., Arkin, N.E., Overstreet, C., et al. (2016). Induced-anxiety differentially disrupts working memory in generalized anxiety disorder. *BMC psychiatry*, **16**, 62

Vytal, K.E., Cornwell, B.R., Arkin, N., et al. (2012). Describing the interplay between anxiety and cognition: From impaired performance under low cognitive load to reduced anxiety under high load. *Psychophysiology*, **49**, 842–52

Vytal, K.E., Cornwell, B.R., Letkiewicz, A.M., et al. (2013). The complex interaction between anxiety and cognition: insight from spatial and verbal working memory. *Frontiers in human neuroscience*, **7**, 93

Yarkoni, T., Poldrack, R., Nichols, T., et al. (2011). NeuroSynth: a new platform for large-scale automated synthesis of human functional neuroimaging data. In: *Frontiers in Neuroinformatics Conference Abstract: 4th INCF Congress of Neuroinformatics*.

**Figure Legends**

**Figure 1: Schematic for experiment. A)** Trials consisted of separate encoding, retention, and response intervals. Low and sort trials contained 5 letter encoding arrays, while high trials contained 8 letter arrays. On low and high trials, subjects retained the letters in the original order, while on sort trials, subjects rearranged the letters in alphabetical order. **B)** Trials were presented during alternating periods of safety and threat. During the safe periods, subjects could not receive a shock. During the threat periods, subjects were informed that they could receive a shock at any time. Trials were shuffled quasi-randomly so that an equal number of each trial type was presented in each block. **C)** Regions of interest for the left (pink) and right (green) dorsolateral prefrontal cortex (dlPFC). An association test for the term “dlPFC” on the site Neurosynth.org [19] was used to generate the bilateral mask.

**Figure 2: Behavioral results. A)** Subjects were more accurate on low trials than on high and sort trials, and less accurate on high than sort trials. **B)** Similarly, subjects showed the fastest reaction time on low trials, and a slower reaction time on high trials than on sort trials. In addition, patients were significantly slower than controls on the high trials. Bars represent means ± SEM. Circles represent individual data points. * pairwise p-value < 0.05.

**Figure 3: Retention interval fMRI ROI results.** Graphs represent significant results from the analysis of the dorsolateral prefrontal cortex region of interest analysis. **A) Load main effect:** Sort trials evoked significantly more activity than high or low trials, and high trials evoked more activity than low trials. **B) Hemisphere main effect:** Activity was greater in the left compared to the right hemisphere. **C) Load x condition interaction:** There was significantly less activity in threat compared to safe during sort trials. **D) Load x hemisphere interaction:** There was significantly less right than left dlPFC activity during sort and low trials. **E) Group x condition interaction:** Patients showed significantly less activity during threat compared to safe trials. **F) Group x hemisphere interaction:** Controls showed significantly less right than left dlPFC activity. Bars represent means ± SEM. Circles represent individual data points. * pairwise p-value < 0.05.

**Figure 4: Correlation between accuracy and right dlPFC activity during the sort condition. A)** Scatterplot showing correlation for controls. B) Scatter plot showing correlation for patients. Lines represent linear trend between accuracy and dlPFC activity. * p-value < 0.05.
